# Circulating tumor DNA in gastrointestinal cancers: promise, pitfalls, and the path forward

**DOI:** 10.1016/j.eclinm.2026.104122

**Published:** 2026-07-31

**Authors:** Sarbajit Mukherjee, Azza Sarfraz, Kyaw Lin Aung, Manmeet Singh Ahluwalia

**Affiliations:** a Medical Oncology, Miami Cancer Institute, Baptist Health South Florida, Miami, FL, USA; b Translational Medicine, Herbert Wertheim College of Medicine, Florida International University, Miami, FL, USA

**Keywords:** Circulating tumor DNA, Minimal residual disease, Gastrointestinal cancers, Colorectal cancer, Liquid biopsy, Precision oncology

## Abstract

Circulating tumor DNA (ctDNA) has emerged as a powerful prognostic biomarker in gastrointestinal (GI) oncology, with the strongest evidence in colorectal cancer. Postoperative ctDNA positivity identifies patients at high risk of recurrence, and serial clearance patterns further refine risk. However, prognostic validity has not consistently translated into treatment-selection utility. In stage II colon cancer, DYNAMIC supported chemotherapy de-escalation without compromising recurrence-free survival, whereas DYNAMIC-III showed that escalation in ctDNA-positive stage III disease did not improve outcomes. More recent randomized data from CIRCULATE suggest that ctDNA-positive patients with proficient mismatch repair and microsatellite-stable stage II colon cancer may benefit from adjuvant chemotherapy, although the intention-to-treat primary endpoint was not statistically significant. COBRA further highlights the vulnerability of low-risk populations to assay-dependent interpretation and unvalidated clearance endpoints. Outside colorectal cancer, ctDNA is strongly prognostic in gastro-esophageal, pancreatic, and biliary tract cancers, but evidence supporting ctDNA-directed treatment remains limited. Assay heterogeneity, variable tumor shedding, postoperative sampling timing, false-positive and false-negative results, and uncertain benefit from treating molecular recurrence before radiographic disease remain major barriers. Prospective trials must show that biomarker-guided interventions improve patient-important outcomes before ctDNA can serve as a stand-alone treatment mandate across GI cancers.

## From prognostic signal to clinical utility

**Fig. 1:**
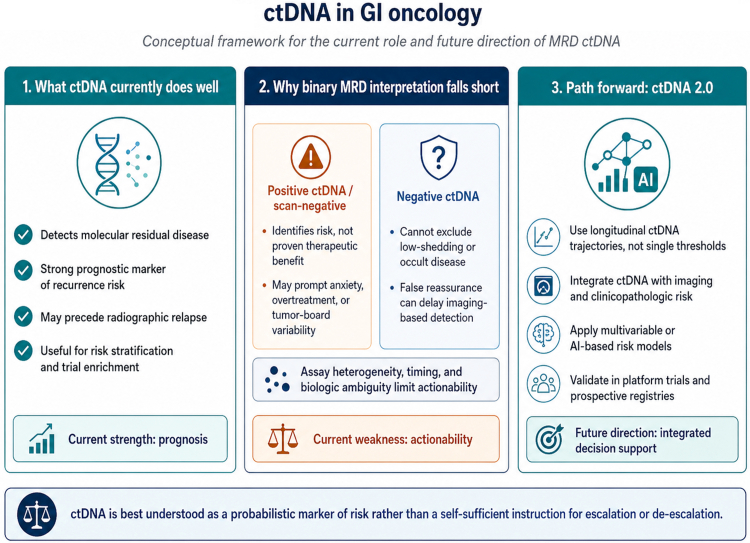
**Conceptual framework for the current role and future direction of MRD ctDNA in GI oncology**. MRD, minimal residual disease; ctDNA, circulating tumor DNA; GI, gastrointestinal.

Circulating tumor DNA (ctDNA) has emerged as a useful tool in gastrointestinal (GI) oncology, particularly as a noninvasive approach for longitudinal disease monitoring.^1^ Because ‘ctDNA reflects tumor-derived genomic alterations in real time, it can track clonal evolution under treatment pressure.^2^ As a short-lived analyte released into the bloodstream from tumor cells, ctDNA broadly correlates with tumor burden, and its detectability is influenced by disease stage, metastatic site, and assay design.^3^ Tumor-informed platforms generally offer greater analytical sensitivity for minimal residual disease (MRD) detection, whereas tumor-agnostic assays may better capture broader genomic evolution and newly emerging resistance mechanisms.^4^ Large prospective observational studies confirm that postoperative ctDNA positivity is strongly prognostic for recurrence and survival, and that ctDNA clearance patterns further refine risk.^5^^,^^6^ Until recently, however, randomized evidence that treatment modification based on MRD improves outcomes was lacking. Emerging colorectal cancer data now suggest that this evidentiary stage is changing, although the strength and maturity of evidence remain disease- and context-specific.^7^ Thus, the central question is no longer only whether ctDNA identifies biologic risk, but when and in whom ctDNA-directed intervention can reproducibly translate that information into better patient outcomes (Figure 1).

## Colorectal cancer as the strongest evidence base

Colorectal cancer provides the strongest evidence base for ctDNA-guided MRD management, and it is therefore the setting in which the transition from prognostic strength toward potential treatment-selection utility is most advanced. In stage II colon cancer, DYNAMIC showed that a ctDNA-guided strategy reduced adjuvant chemotherapy use from 28% to 15% without compromising 2-year recurrence-free survival, thereby supporting selective de-escalation.^8^ However, the trial did not directly establish benefit for ctDNA-positive patients from chemotherapy, let alone from ctDNA-guided escalation, because ctDNA-positive patients were not randomized to chemotherapy versus observation.^8^ That limitation was not merely semantic; DYNAMIC was a phase II noninferiority study with a wide 8.5 percentage-point margin, was powered on recurrence-free survival (RFS) rather than overall survival, and resulted in substantially greater oxaliplatin use in the ctDNA-guided arm, 9.5% versus 2.7%, without evidence of improved outcomes.^9^ Findings from the DYNAMIC trial support chemotherapy sparing in selected patients, but do not validate postoperative ctDNA positivity as a mandate for intensified adjuvant therapy.^9^

The 2026 CIRCULATE AIO-KRK-0217/ABCSG trial provides the first prospective randomized signal that postoperative ctDNA positivity may identify patients with stage II proficient mismatch repair/microsatellite-stable colon cancer who may benefit from adjuvant chemotherapy.^7^ Among ctDNA-positive patients, the per-protocol analysis showed improved 3-year disease-free survival (DFS) with chemotherapy versus observation, 77% versus 38%, and reduced 3-year recurrence, 19% versus 62%.^7^ However, the intention-to-treat primary endpoint was not statistically significant, with 3-year DFS of 61% versus 38%; interpretation is limited by premature trial closure, small sample size, treatment nonadherence, and reliance on the per-protocol analysis.^7^ These findings extend ctDNA beyond purely prognostic risk assessment in colorectal cancer and support potential treatment-selection utility, but they do not establish a universal or assay-independent treatment mandate.

That distinction became even clearer in stage III disease. DYNAMIC-III confirmed that postoperative ctDNA is strongly prognostic, with 3-year RFS of 87% in ctDNA-negative patients versus 49% in ctDNA-positive patients.^10^ Yet that prognostic separation did not translate into therapeutic benefit from escalation. In ctDNA-negative patients, de-escalation reduced oxaliplatin use from 88.6% to 34.8% and treatment-related hospitalization from 13.2% to 8.5%, but 3-year RFS was 85.3% versus 88.1% and did not meet the prespecified noninferiority margin.^10^ In ctDNA-positive patients, escalation failed to improve 2-year RFS, 51% versus 61%, despite a marked gradient of worsening prognosis with increasing ctDNA burden and a 3-year RFS of only 14% in patients with persistent ctDNA after treatment.^10^ Taken together, DYNAMIC-III validated ctDNA as a powerful marker of postoperative risk but not as a clinically effective guide for treatment intensification.^10^

Findings from the COBRA trial add a different but equally important caution because it tested ctDNA-guided escalation in the setting where assay interpretation is most vulnerable, namely low-risk stage II colon cancer. In the preplanned phase II analysis, ctDNA clearance at 6 months was observed in three (43%) of seven patients assigned to surveillance and in 1 (11%) of 9 patients assigned to adjuvant FOLFOX or CAPOX, leading to early study closure under the prespecified futility rules.^11^ The significance of COBRA lies less in the negative comparison itself than in what it revealed about assay-dependent interpretation in a low-risk population. ctDNA clearance was used as the phase II endpoint even though it is not yet a validated surrogate for disease-free survival (DFS) or overall survival, and the study was conducted in stage II disease, where the low baseline recurrence risk makes assay specificity and positive predictive value central to interpretation.^6^ In that setting, the unexpectedly high ctDNA clearance rate with surveillance, 43%, and the low clearance rate with chemotherapy, 11%, contrasted with prior data, including spontaneous clearance rates of 12.2% and chemotherapy-associated clearance rates of 68.5% in GALAXY, raising the possibility that assay limitations, biologic fluctuation around the detection threshold, or false-positive classification influenced the result.^12^^,^^13^ For instance, in a population with an approximately 85% recurrence-free rate, even an assay specificity of 95% could still generate a clinically meaningful false-positive burden, highlighting the importance of disease prevalence and positive predictive value when interpreting ctDNA positivity in low-risk populations.^14^, ^15^, ^16^, ^17^ COBRA therefore did not invalidate ctDNA as a prognostic biomarker; rather, it showed that postoperative ctDNA positivity in low-risk stage II disease is not yet a sufficiently robust therapeutic trigger for routine escalation, and that assay performance, endpoint validity, and disease context must be defined more precisely before ctDNA-guided intensification can be considered clinically reliable.

The same gap between prognostic information and clinically meaningful benefit is evident in surveillance. In a real-world cohort of resected stage II to IV colorectal cancer, serial ctDNA surveillance was prognostically informative but added little incremental curative value beyond guideline-concordant imaging.^18^ Although 20 patients had ctDNA-positive, imaging-negative recurrence at first detection, only three (1.6%) of 184 patients ultimately had durable disease-free status attributable to ctDNA-triggered early intervention, whereas 14 recurrences were detected by imaging despite negative ctDNA and three ctDNA-positive patients cleared without radiographic recurrence.^18^ These findings suggest that earlier molecular detection alone does not necessarily translate into a meaningful increase in durable salvage over high-quality standard surveillance.

## Evidence beyond colorectal cancer

In GI malignancies other than colorectal cancer, the prognostic value of ctDNA has outpaced its therapeutic utility.^19^^,^^20^ In gastro-esophageal adenocarcinoma, ctDNA detected after neoadjuvant therapy or surgery is associated with worse disease-specific survival and shorter progression-free survival. However, assay sensitivity remains limited in localized disease.^19^ In one resected esophageal adenocarcinoma cohort, 66% of patients without detectable postoperative ctDNA still died of disease by 40 months, consistent with the known low-shedding biology of localized esophageal adenocarcinoma.^19^ Across GI cancers, technical strategies under evaluation include tumor-informed mutation tracking, plasma-only targeted next-generation sequencing, digital PCR, and tissue-free approaches incorporating genomic, methylation, or combined genomic-epigenomic signals.^19^^,^^21^^,^^22^ Methylation-based approaches may improve detection because cancer-associated differentially methylated regions can be more recurrent and tissue-specific than individual mutations; a pan-GI cell-free DNA methylation platform demonstrated an area under curve of 0.90 for gastric cancer detection while also supporting tissue-of-origin classification.^22^ Fragmentomic approaches interrogate genome-wide fragment size, end motifs, nucleosome positioning, copy-number patterns, and chromatin organization, potentially increasing signal recovery when variant allele fractions are low.^23^ By integrating these complementary features with genomic alterations, such assays may improve patient stratification by refining recurrence-risk estimates, identifying the likely tissue of origin, and informing surveillance intensity or enrollment in biomarker-directed trials. However, prospective validation is required before these complex signatures can be used to guide treatment decisions.^19^

Pancreatic ductal adenocarcinoma illustrates the same limitation in a biologically more aggressive disease.^24^ In resected pancreatic ductal adenocarcinoma, perioperative tumor-informed ctDNA positivity in the MRD window was associated with a median DFS of 6.4 months compared with 33.3 months in ctDNA-negative patients.^24^ During surveillance, ctDNA positivity was associated with a hazard ratio (HR) of 12.4 for recurrence, and in multivariable analysis it was the strongest prognostic factor, with an HR of 24.3.^24^ These data indicate that ctDNA identifies patients at very high risk of early relapse, but they do not show that ctDNA-directed treatment adaptation alters that outcome.^20^ Recent evidence in pancreatic ductal adenocarcinoma therefore frames MRD ctDNA as promising for perioperative risk stratification, but it still requires prospective validation before implementation.^20^

In cholangiocarcinoma, the interpretative challenge is compounded by tumor biology itself.^25^ ctDNA release is lower than in colorectal cancer because of dense stromal fibrosis and sparse vascular distribution.^25^ Current applications remain largely exploratory, testing is not standardized, and tumor-naive assays are more likely to be negative when tumor burden is low.^25^ Accordingly, ctDNA in cholangiocarcinoma currently serves more convincingly as a tool for prognostic stratification and molecular profiling than as a validated MRD-guided treatment strategy.^25^

## Technical and clinical limitations

These disease-specific limitations are mirrored by broader technical limitations of the assays themselves. Assay heterogeneity remains a major barrier to routine MRD interpretation. Tumor-informed and tumor-agnostic platforms differ in tissue requirements, turnaround time, detection thresholds, serial sensitivity, and susceptibility to false positives. Analytical detection thresholds vary across platforms, with some tumor-informed assays capable of detecting variant allele fractions near 0.01%, whereas several tumor-agnostic approaches operate at higher detection thresholds; serial sensitivity has been reported at 88% versus 59%, respectively.^26^ MRD classification therefore remains assay-dependent rather than fully standardized.^26^ Across major colorectal cancer datasets, reported ctDNA clearance has ranged from 11% to 68.5%, underscoring that clearance remains highly context-dependent and not yet sufficiently standardized to serve as a stand-alone treatment-directing endpoint.^26^ Postsurgical studies also indicate that ctDNA behaves as a quantitative continuum, with recurrence risk increasing as mutant allele fraction rises rather than conforming to a purely binary biologic state.^27^ Postoperative ctDNA detection in pooled colorectal cancer cohorts was 11.0% at 4–6 weeks, 12.5% at 6–8 weeks, and 13.8% at 8–10 weeks, while ctDNA half-life in postoperative studies ranged from 35 to 139 min across tumor types.^27^^,^^28^ The biologic signal is therefore timing-dependent, and no single postoperative window has been fully standardized for treatment-directing MRD assessment.^27^^,^^28^

These limitations become most consequential when ctDNA is acted upon in routine care without a validated response framework. When ctDNA is used in the MRD setting without a validated response strategy, it can generate risk information that exceeds its established clinical utility.^29^ A positive, scan-negative result identifies recurrence risk, but it does not establish that earlier treatment improves disease-free or overall survival.^29^ It remains uncertain whether initiating systemic therapy in the absence of radiographically evident disease provides meaningful clinical benefit, and ctDNA positivity before radiologic relapse may itself carry psychological consequences.^29^ Conversely, current MRD assays can miss patients who later recur, particularly in low-shedding settings, and negative ctDNA cannot replace imaging-based surveillance because up to half of patients who eventually recur can initially test negative on current MRD assays.^29^ Adding serial ctDNA to National Comprehensive Cancer Network-guided imaging yielded durable disease-free status attributable to ctDNA-triggered intervention in only 1.6% of patients in one real-world cohort.^18^ Longitudinal colorectal cancer cohorts similarly demonstrate that recurrence can still occur among ctDNA-negative patients.^26^^,^^30^ Further, ctDNA implementation depends on assay cost, reimbursement, tumor-tissue availability for tumor-informed platforms, turnaround time, sequencing infrastructure, data systems, and specialized interpretation. Even favorable economic estimates, including a projected average saving of $9771, remain model-based rather than outcome-proven.^26^^,^^30^

## The path forward for ctDNA 2.0

We propose a more rigorous ‘ctDNA 2.0’ model based on serial trajectories and multimodal liquid-biopsy signals rather than isolated postoperative threshold. ctDNA findings should be interpreted alongside imaging, clinicopathologic risk factors, metastatic pattern, treatment timing, and assay characteristics, and may ultimately be incorporated into multivariable or artificial intelligence–based models that dynamically estimate recurrence risk over time.^29^ Genomic alterations may be integrated with methylation and fragmentomic features to improve analytical sensitivity and tissue-of-origin inference while circulating tumor cells, extracellular vesicles, circulating RNA, and protein biomarkers may provide complementary information regarding viable disease, tumor phenotype, treatment resistance, and the tumor microenvironment.^6^^,^^21^, ^22^, ^23^ Such multimodal integration may overcome the limitations of any single analyte; however, its clinical implementation will require standardized preanalytic procedures, prospectively calibrated models, prespecified decision thresholds, and evidence that biomarker-guided interventions improve patient outcomes.

The National Cancer Institute GI Oncology ctDNA Working Group emphasizes that ctDNA-guided treatment strategies require prospective validation and that ctDNA is not yet validated as a surrogate endpoint for progression-free survival or overall survival in late-phase GI trials.^29^ Colorectal cancer is now an important exception in evidentiary trajectory. Emerging randomized data in stage II proficient mismatch repair/microsatellite-stable colon suggest that ctDNA positivity may identify patients who benefit from adjuvant chemotherapy, although the statistically nonsignificant intention-to-treat result and trial limitations preclude treating this signal as definitive or universally generalizable.^7^ Other GI cancers remain largely at the prognostic-validation stage. Accordingly, ctDNA should not yet be regarded as a universal stand-alone treatment mandate across GI cancers; rather, its clinical role should be defined by tumor type, disease stage, assay platform, and the maturity of prospective interventional evidence. Ongoing platform trials and prospective registries remain essential to determine whether longitudinal ctDNA-guided strategies improve outcomes that matter to patients.^29^

## Contributors

S.M. and A.S. conceptualized the manuscript. S.M. and A.S. performed the literature search and drafted the original manuscript. A.S. contributed to manuscript organization, reference review, and figure development. M.S.A. and K.L.A. provided critical intellectual input, supervision, and writing–review and editing. All authors contributed to interpretation of the published literature discussed in this Viewpoint and critically revised the manuscript. All authors read and approved the final version of the manuscript. S.M. and A.S. had access to and verified the accuracy of the cited literature and were responsible for the decision to submit the manuscript.

## Declaration of interests

S.M. has received research funding from the National Comprehensive Cancer Network and Ipsen Biopharmaceuticals/North American Neuroendocrine Tumor Society (NANETS), paid to the institution and outside the submitted work; has received consulting fees from Astellas, Bristol Myers Squibb (BMS), BeOne Ltd, Boehringer Ingelheim, Eisai, Exelixis, Gilead, Incyte, Jazz Pharmaceuticals, Merck, Natera, and Sirtex; and has received payment or honoraria for lectures, presentations, or educational events from Meetings Events & Conference Coordinators, Binaytara Foundation, Total Health, and the Florida Society of Clinical Oncology. S.M. serves as a board member of the Esophageal Cancer Action Network and as a guidelines panel member for the International Society for Diseases of the Esophagus, American Society of Clinical Oncology, North American Neuroendocrine Tumor Society, and National Comprehensive Cancer Network. M.S.A. has received grants or contracts from Pfizer and Incyte, paid to the institution; has received consulting fees from Bayer AG Pharmaceuticals, Xoft, Nuvation Bio, Apollomics, ViewRay, Varian Medical Systems, Theraguix, AnHeart Therapeutics, Menarini Ricerche, Sumitomo Pharma Oncology, Autem Therapeutics, GT Medical Technologies, Modifi Biosciences, Bugworks, AlloVir, EquilliumBio, VBI Vaccines, Servier Pharmaceuticals, Incyte, Recordati, Genmab, and Novocure; and holds stock or stock options in Mimivax, Modifi Biosciences, TriSalus Life Sciences, LiveAI, and Ryght. A.S. and K.L.A. declare no competing interests.
